# Progress Toward Eradication of Dracunculiasis (Guinea Worm Disease) — Worldwide, January 2024–June 2025

**DOI:** 10.15585/mmwr.mm7442a2

**Published:** 2026-01-01

**Authors:** Donald R. Hopkins, Adam J. Weiss, Sarah Yerian, Ynes R. Ortega, Yujing Zhao, Obiora A. Eneanya, Vitaliano A. Cama

**Affiliations:** ^1^Guinea Worm Eradication Program, The Carter Center, Atlanta, Georgia; ^2^Center for Food Safety, College of Agriculture & Environmental Sciences, University of Georgia, Griffin, Georgia; ^3^Division of Foodborne, Waterborne, and Environmental Diseases, National Center for Emerging and Zoonotic Infectious Diseases, CDC.

**Summary**

**What is already known about this topic?**

During 1986–2023, human dracunculiasis cases decreased by >99%, from an estimated 3.5 million to 14 worldwide. Since 2012, the transmission of dracunculiasis in dogs has complicated eradication efforts.

**What is added by this report?**

Fifteen human dracunculiasis cases and 664 animal infections were reported worldwide in 2024, and one human case and 550 animal infections were reported during January–June 2025. As of June 2025, indigenous dracunculiasis transmission was occurring in six countries (Angola, Cameroon, Chad, Ethiopia, Mali, and South Sudan).

**What are the implications for public health practice?**

Program efforts have brought dracunculiasis close to eradication. However, animal infections and public health personnel’s impeded access to the population due to civil unrest and insecurity in Mali, South Sudan, and Sudan are the most important remaining programmatic challenges.

## Abstract

Dracunculiasis (Guinea worm disease), caused by the parasite *Dracunculus medinensis*, is acquired by drinking water containing small water fleas infected with *D. medinensis* larvae or eating inadequately cooked aquatic animals. Efforts to eradicate *D. medinensis*, including the Guinea Worm Eradication Program (GWEP), began at CDC in 1980. In 1986, with an estimated 3.5 million cases in 20 African and Asian countries, the World Health Assembly called for dracunculiasis elimination in specific geographic areas; this goal was later expanded to global eradication. GWEP has been led by The Carter Center since 1986 and is supported by countries with endemic dracunculiasis, CDC, the World Health Organization, UNICEF, and other partners. During 1986–2023, human dracunculiasis cases decreased by >99%, from an estimated 3.5 million to 14 worldwide. Since 2012, environmental contamination from infected animals has posed a new challenge to eradication, as have ongoing civil unrest and insecurity in some areas. As of June 2025, indigenous dracunculiasis transmission was occurring in six countries (Angola, Cameroon, Chad, Ethiopia, Mali, and South Sudan). Fifteen human cases and 664 animal infections were reported in 2024, including 299 canine infections in Cameroon and 234 in Chad; during January–June 2025, one human case and 550 animal infections were reported. Animal infections and public health personnel’s impeded access to the population due to civil unrest and insecurity in Mali, South Sudan, and Sudan threaten the near-term possibility of disease eradication. Nevertheless, countries and partners appear poised to reach zero human cases soon.

## Introduction

Dracunculiasis (Guinea worm disease), caused by the parasite *Dracunculus medinensis*, is acquired by drinking water containing small copepods (water fleas) infected with *D. medinensis* larvae (*1*) or eating inadequately cooked aquatic animals such as fish or raw fish entrails (*2*). Eradication efforts and the Guinea Worm Eradication Program (GWEP) were initiated by CDC in 1980 (*1*). Backers of the International Drinking Water Supply and Sanitation Decade endorsed the Guinea worm eradication initiative in 1981; the World Health Organization (WHO) made CDC a WHO Collaborating Center for Guinea Worm in 1984; and CDC, WHO, and the United States Agency for International Development convened the first international meeting on Guinea worm eradication in 1982. In 1986, with 3.5 million reported human cases* in 20 countries^†^ (*3*), the World Health Assembly (WHA) initially called for dracunculiasis elimination (i.e., within specific geographic areas), and this goal was later expanded to global eradication. The aim of GWEP is elimination of Guinea worm from all countries in the world. Led by The Carter Center and supported by CDC, WHO, UNICEF, and other partners, GWEP assists ministries of health in countries with dracunculiasis. During 1986–2023, human dracunculiasis cases decreased by >99%, from an estimated 3.5 million to 14 worldwide. Since 2014, GWEP has also used a cash reward system for laboratory-confirmed Guinea worm infections to increase reporting sensitivity (*4*). This system offers a small sum of money to persons reporting worms that have emerged through the skin (hanging worms) and are laboratory-identified as *D. medinensis*.

Guinea worm eradication relies on case containment^§^ and on prevention of environmental contamination by infected dogs, which plays an important role in sustaining transmission to humans, cats, and other dogs (*1*)*.* Since 2018, proactive tethering of all dogs has been recommended in communities at high risk or with high endemicity during the peak transmission season to prevent both the dogs’ exposure to contaminated sources and contamination of water by infected dogs (*5*,*6**)*. Additional interventions include health education, water filtration or treatment with the organophosphate larvicide temephos, provision of safe drinking water, adequate cooking of aquatic animals, and safe disposal of fish entrails (*1*,*2*). WHO has certified 200 countries and territories as dracunculiasis-free. In addition to Sudan, which has not completed certification because of civil insecurity, six countries with ongoing dracunculiasis remain (Dracunculiasis Eradication: Global Surveillance Summary, 2023 | WHO). Since 2012, eradication efforts have been challenging because of animal infections, mostly in dogs, especially in Cameroon, Chad (*7*,*8*), and Angola (Dracunculiasis Eradication: Global Surveillance Summary, 2022 | WHO). This report updates previous reports and describes progress during January 2024–June 2025 (Dracunculiasis Eradication: Global Surveillance Summary, 2024 | WHO) (*5*).

## Methods

### Country Reports

Each country’s GWEP provided data collected on *D. medinensis* infections during January 2024–June 2025, based on monthly records of human cases and animal infections, containment of Guinea worm infections, availability of safe drinking water, and Guinea worm educational outreach. Programs receive monthly reports from volunteer supervisors in villages.^¶^ Specimens requiring laboratory confirmation are sent to CDC, and, since August 2024, also to the University of Georgia. Villages remain under surveillance for 3 years after the last known infection, and all data are compiled and maintained by the country’s ministry of health GWEP. This activity was reviewed by CDC, deemed not research, and was conducted consistent with applicable federal law and CDC policy.**

### WHO Certification of Eradication

WHO certifies a country that has adequate nationwide surveillance to be dracunculiasis-free after ≥3 consecutive years with no indigenous infection.^††^ Eradication status of reporting countries was compiled and reported. 

## Results

### Human Cases and Animal Infections

During 2024, a total of 15 human dracunculiasis cases were identified worldwide, including nine in Chad and six in South Sudan (Table 1), one more than the 14 total human cases reported in 2023 (Table 2). One case was reported during January–June 2025, compared with three during the same period in 2024. A total of 664 animal infections were reported from Angola, Cameroon, Chad, Ethiopia, Mali, and South Sudan in 2024, a 22% decrease from the 854 reported in 2023 (Table 2). The 550 animal infections reported during January–June 2025 represent a 20% increase compared with the 459 reported during the same period in 2024. Among the 664 animal infections reported during 2024, 591 (89%) were reported by Cameroon (310; 47%) and Chad (281; 42%). Among 550 animal infections reported during January–June 2025, 478 (87%) were reported by these two countries (Cameroon: 398; 72%, and Chad: 80; 15%).

**TABLE 1:** Reported dracunculiasis human cases and animal infections, surveillance, and status of local interventions in villages with endemic disease, by country — worldwide, 2024

Characteristic	Country						
	Angola	Cameroon	Chad*	Ethiopia	Mali^†^	South Sudan	Total
**Reported human cases**							
Indigenous, no.	0	0	9	0	0	6	**15**
Imported, no.	0	0	0	0	0	0	**0**
Contained,^§^ % (no./total no.)	NA	NA	22 (2/9)^¶^	NA	NA	17 (1/6)	**20 (3/15)**
Change in indigenous human cases in villages/localities under surveillance, same period in 2023 and 2024, % (2023 to 2024)	NA (0 to 0)	−100 (1 to 0)	0 (9 to 9)	NA (0 to 0)	−100 (1 to 0)	200 (2 to 6)	15** (13 to 15)
**Reported animal infections**							
Indigenous, no.	39	310	281	2	28	4	**664**
Imported, no.	0	0	0	0	0	0	**0**
Contained,^§^ % (no./total no.)	28 (11/39)	74 (230/310)	67 (188/281)	0 (0/2)	54 (15/28)	75 (3/4)	**67 (447/664)**
Change in indigenous human cases in villages/localities under surveillance, same period in 2023 and 2024, % (2023 to 2024)	−55 (87 to 39)	38 (224 to 310)	−43 (496 to 281)	NA (0 to 2)	−39 (46 to 28)	300 (1 to 4)	**−22 (854 to 664)**
**Villages under active surveillance, no.**	151	111	2,785	388	1,966	2,490	**7,891**
Reporting monthly, %	100	100	100	100	100	96	**99**
≥1 human case	0	0	8	0	0	4	**12**
Only imported human cases	0	0	0	0	0	0	**0**
Indigenous human cases	0	0	8	0	0	4	**10**
≥1 animal infection	21	15	177	1	23	4	**241**
Only imported animal infections	0	0	0	0	0	0	**0**
Indigenous animal infections	21	15	177	1	23	4	**241**
**Status/interventions in villages with endemic human dracunculiasis**							
Villages with endemic human dracunculiasis, 2023–2024, no.	0	1	13	0	1	6	**21**
Reporting monthly, % (no./total no.)	NA	100 (1/1)	100 (13/13)	NA	100 (1/1)	100 (6/6)	**100 (21/21)**
Filters in all households, % (no./total no.)	NA	100 (1/1)	92 (12/13)	NA	100 (1/1)	100 (6/6)	**95 (20/21)**
Using temephos, % (no./total no.)	NA	100 (1/1)	85 (11/13)	NA	100 (1/1)	100 (6/6)	**90 (19/21)**
≥1 source of safe water, % (no./total no.)	NA	100 (1/1)	62 (8/13)	NA	100 (1/1)	100 (6/6)	**76 (16/21)**
Provided health education, % (no./total no.)	NA	100 (1/1)	85 (11/13)	NA	100 (1/1)	100 (6/6)	**90 (19/21)**
**Status/interventions in villages with endemic animal dracunculiasis**							
Villages with endemic animal dracunculiasis, 2023–2024, no.	83	16	379	2	40	5	**525**
Reporting monthly, % (no./total no.)	100 (83/83)	100 (16/16)	100 (379/379)	100 (2/2)	100 (40/40)	100 (5/5)	**100 (525/525)**
Using temephos, % (no./total no.)	17 (14/83)	69 (11/16)	86 (325/379)	100 (2/2)	100 (40/40)	100 (5/5)	**76 (397/525)**
Provided health education, % (no./total no.)	100 (83/83)	100 (16/16)	90 (341/379)	100 (2/2)	100 (40/40)	100 (5/5)	**93 (487/525)**

**Abbreviations**: CAR = Central African Republic; GWEP = Guinea Worm Eradication Program; NA = not applicable.

* Participants at the annual Chad GWEP review meeting in November 2014 adopted “1+ case village” as a new description for villages in Chad affected by human cases of Guinea worm disease, dogs infected with Guinea worms, or both, and defined it as a village with one or more indigenous cases of Guinea worm infections, imported cases, or both in humans, dogs, or cats in the current calendar year, previous year, or both.”

^†^ Civil unrest and insecurity since a coup d'état in April 2012 continued to constrain GWEP operations (e.g., supervision, surveillance, and interventions) in regions with endemic dracunculiasis (Gao, Kidal, Mopti, Segou, and Timbuktu) during January 2021–June 2025.

^§^ Human cases are considered contained when all of the following criteria are met: 1) infected patients are identified within 24 hours of worm emergence; 2) patients have not entered any water source since the worm emergence; 3) a village volunteer or health care provider has properly treated the lesion until all detectable worms are fully removed and has educated the patient not to contaminate water sources; 4) the containment process is validated by a GWEP supervisor within 7 days of worm emergence; and 5) all contaminated and potentially contaminated sources of drinking water are treated with temephos. The criteria for defining a contained case of dracunculiasis in a human should also be applied, as appropriate, to define containment for an animal with Guinea worm infection.

^¶^ A total of six human cases were reported from Chad in 2022, and nine in 2023. One human case was reported from CAR in 2022 and one in 2023. These two human cases might have been acquired in Chad.

** Excluding one human case in CAR.

**TABLE 2:** Number of reported indigenous human and animal dracunculiasis cases, by country — worldwide, January 2023–June 2025

Characteristic	Country						
	Angola	Cameroon*	Chad^†^	Ethiopia	Mali^§^	South Sudan	Total
**Human cases, no. (% contained)^¶^**							
Jan–Dec 2023	0 (—)	1 (100)	9 (67)	0 (—)	1 (0)	2 (0)	13 (54)**
Jan–Dec 2024	0 (—)	0 (—)	9 (22)	0 (—)	0 (—)	6 (17)	**15 (63)**
Change from Jan–Dec 2023 to Jan–Dec 2024, %	NA	−100	0	NA	–100	200	**15**
Jan–Jun 2024	0 (—)	0 (—)	1 (0)	0 (—)	0 (—)	2 (50)	**3 (33)**
Jan–Jun 2025	0 (—)	0 (—)	1 (0)	0 (—)	0 (—)	0 (—)	**1 (0)**
Change from Jan–Jun 2024 to Jan–Jun 2025, %	NA	NA	0	NA	NA	−100	**−67**
**Animal infections,** ^††^ ** no. (% contained)^¶^**							
Jan–Dec 2023	87 (2)	224 (87)	496 (76)	0 (—)	46 (76)	1 (0)	**854 (72)**
Jan–Dec 2024	39 (28)	310 (74)	281 (67)	2 (0)	28 (54)	4 (75)	**664 (63)**
Change from Jan–Dec 2023 to Jan–Dec 2024, %	−55	38	−43	NA	−39	300	**−22**
Jan–Jun 2024	36 (28)	279 (74)	144 (65)	0 (—)	0 (—)	0 (—)	**459 (68)**
Jan–Jun 2025	70 (43)	398 (74)	80 (71)	0 (—)	2 (0)	0 (—)	**550 (69)**
Change from Jan–Jun 2024 to Jan–Jun 2025, %	94	43	−44	NA	NA	NA	**20**

**Abbreviations:** CAR = Central African Republic; GWEP = Guinea Worm Eradication Program; NA = not applicable.

* One human case and multiple animal infections detected in areas of Cameroon near the border with Chad might have been infected in Chad. Cameroon has 117 provisional dog infections and eight provisional cat infections, for which laboratory confirmation is pending.

^†^ Chad’s human case counts for January–December 2022 and January–December 2023 each include one human case detected in an area of CAR.

^§^ Civil unrest and insecurity since a coup d'état in April 2012 continued to constrain GWEP operations (supervision, surveillance, and interventions) in regions with endemic dracunculiasis (Gao, Kidal, Mopti, and Timbuktu) during 2021–June 2025.

^¶^ Human cases are contained when all of the following criteria are met: 1) infected patients are identified within 24 hours of worm emergence; 2) patients have not entered any water source since the worm emergence; 3) a village volunteer or health care provider has properly treated the lesion until all detectable worms are fully removed and has educated the patient not to contaminate water sources; 4) the containment process is validated by a GWEP supervisor within 7 days of worm emergence; and 5) all contaminated and potentially contaminated sources of drinking water are treated with temephos. The criteria for defining a contained case of dracunculiasis in a human should also be applied, as appropriate, to define containment for an animal with Guinea worm infection.

** Excluding one human case in CAR.

^††^ In Chad, primarily dogs, some cats; in Ethiopia, dogs, cats, and baboons; in Mali, dogs and cats; in Angola, dogs; in Cameroon, dogs and cats.

### Laboratory Analysis of Specimens

During January–June 2025, CDC received seven worm specimens from humans; three of these were laboratory confirmed as *D. medinensis* (Table 3),^§§^ compared with one of seven human specimens confirmed as *D. medinensis* during January–June 2024. During January–June 2025, CDC and the University of Georgia received 731 worm specimens from animals, 663 (91%) of which were laboratory confirmed *D. medinensis*, compared with 494 (92%) confirmed worm specimens from among 545 received during January–June 2024.

**TABLE 3:** Characteristics of human and animal worm specimens* received at CDC and the University of Georgia for laboratory diagnosis of *Dracunculus medinensis* — worldwide, January 2024–June 2025

Characteristic	Years/Months			
	2025	2024		
	Jan–Jun	Jan–Jun	Jul–Dec	Jan–Dec
**Total no. of human specimens**	**7**	**7**	**34**	**41**
Positive specimens^†^/no. specimens received (no. of patients), by country of origin				
Chad	3/3 (3)	1/3 (1)	10/11 (8)	11/14 (9)
Ethiopia	—^§^	0/1 (0)	—	0/1 (0)
Kenya (postelimination surveillance)	—	0/1 (0)	—	0/1 (0)
South Sudan	—	0/2 (0)	9/22 (6)	9/24 (6)
Sudan	—	—	0/1 (0)	0/1 (0)
Total positive, no. (%)	3 (43)	1 (14)	19 (56)	20 (49)
Negative specimens,^†^ by other laboratory identifications, no. (%)				
*Onchocerca* species	—	2 (29)	—	2 (10)
Other parasitic nematode^¶^	1 (14)	1 (14)	5 (33)	6 (29)
Sparganum	1 (14)	1 (14)	5 (33)	6 (29)
Other parasitic cestode	1	—	—	—
Tissue (animal origin)	2 (28)	1 (14)	4 (27)	5 (24)
Unknown origin**	—	1 (14)	1 (7)	2 (10)
Total negative, no. (%)	4 (57)	6 (86)	15 (44)	21 (51)
**Total no. of animal specimens**	**731**	**545**	**540**	**1,085**
Positive specimens^†^ by country and species of origin, no. of specimens (no. of animals)*				
Angola				
Dog	129 (63)	50 (50)	—	50 (50)
Cameroon				
Cat	23 (18)	10 (5)	9 (7)	19 (11)
Dog	461 (250)	420 (209)	174 (137)	594 (310)
Chad				
Cat	4 (4)	—	—	—
Dog	35 (20)	11 (8)	5 (5)	16 (13)
Other animal (monkey)	—	—	1 (1)	1 (1)
Ethiopia				
Baboon	8 (2)	1 (1)^††^	3 (3)	4 (4)
Mali				
Cat	—	—	6 (6)	6 (6)
Dog	2 (2)	—	23 (22)	23 (22)
Other animal (jackal)	—	—	2 (1)	2 (1)
South Sudan				
Cat	1 (1)	—	2 (2)	2 (2)
Dog	—	—	1 (1)	1 (1)
Civets or genets (Viverridae family)	—	—	7 (7)	7 (7)
Other animals (wildcats)	—	2 (2)	8 (8)	10 (10)
Positive,^† ^no. (%)	663 (91)	494 (92)	258 (48)	752 (69)
Total negative,^†^ no. (%)	68 (9)^§§^	51 (8)	282 (52)	333 (31)^¶¶^

* Specimen is defined as each presumed worm that emerged through the skin (hanging worm) submitted for laboratory confirmation of *Dracunculus medinensis*.

^†^ Positive specimens were confirmed as *D. medinensis*; negative specimens ruled out as *D. medinensis*.

^§^ Dashes indicate no specimen was received.

^¶^ Other parasitic nematodes submitted in association with human cases were identified as follows: four belonging to the Mermithidae family, one to the Ascarididae family, and one to the Nematoda phylum.

** Specimens microscopically ruled out as *D. medinensis* but without features that allow further identification.

^††^ Subcutaneous worms not yet emerged extracted from a dead baboon. Worms that have not emerged do not meet the case definition and are not counted as infections.

^§§^ In 2025, four submissions with degraded material beyond recognition and 64 negative specimens were identified as follows: 28 were spargana, 21 were other parasitic nematodes: six belonging to the Filariidae family, four to the Diplotriaenidae family, and four to the Nematoda phylum; three were *Setaria* spp., two were *Physaloptera* spp., and one was a *Tanqua* spp.; and one belonging to the Ascarididae family. A total of 28 were spargana, 13 were animal tissues, one was plant tissue, and one was an *Acantocephala* spp.

^¶¶^ In 2024, the 333 negative specimens were identified as follows: 142 were other parasitic nematodes: 38 belonging to the Nematoda phylum, 33 to the Diplotraenidae family, 23 to the Filariidea family, and 10 to the Ascarididae family; nine were *Tanqua* spp. and six were *Dracunculus* spp. (not *D. medinensis);* five belonged to the Mermithidae family and four to the Spiruridae family; three were *Physaloptera* spp. and three were *Setaria* spp.; two belonged to the Chromadorea class, two to the Dracunculidae family, and one to the Angiostrogyliidae family; and one was a *Dirofilaria* sp. A total of 93 were spargana, 59 were tissues of animal origin, nine were other parasitic cestodes, two were free-living organisms, one was plant material, and four were unidentifiable wormlike or stringlike sections of unknown origin.

### Country Reports

**Angola.** No human cases were detected in the 151 communities under surveillance in Angola in 2024 (Table 1). Whereas 39 infected dogs were detected during all of 2024, 70 such infections were detected during January–June 2025 (Table 2), a 79% increase. Genetic analysis has not identified a direct link between Angola’s *D. medinensis* and specimens from other countries (E Thiele, PhD, Vassar College, personal communication, August 2025). Angola uses temephos in affected areas and in 2024 started preparations to tether dogs at risk for infection.

**Cameroon.** Cameroon detected Guinea worm in 2019 after having reported no cases since 1997 and being certified Guinea worm–free by WHO in 2007. Guinea worm was initially imported from adjacent areas of Chad, and indigenous transmission was reestablished in Cameroon within a few years. Cameroon reported no human cases in 2024 or during January–June 2025. In 2024 and during January–June 2025, a total of 310 (Table 1) and 398 (Table 2) infected animals, respectively, were reported in 20 villages close to the Chad-Cameroon border. Cameroon expanded active surveillance by training local village volunteers and their supervisors, while implementation of their policy for tethering dogs^¶¶^ reached 79% compliance.

**Chad**. Chad reported nine human cases in both 2023 and 2024, and one case during both January–June of 2024 and 2025 (Table 2). Chad reported 43% fewer animal infections in 2024 (281) than in 2023 (496) and 44% fewer infected animals during January–June 2025 (80) than during January–June 2024 (144). By December 2024, Chad had implemented surveillance in 2,785 villages (Table 1). In areas with established surveillance, 62% and 55% of residents surveyed during 2024 and January–June 2025, respectively, were aware of the rewards for reporting a case of dracunculiasis. In villages reporting dog infections during the preceding or current year, proactive tethering of eligible dogs reached 70% and 45% during 2024 and January–June 2025, respectively.

Water treatment with temephos reached all 184 villages with reported dracunculiasis by December 2024; by June 2025, temephos treatment reached 225 villages that either reported dracunculiasis or were at high risk for dracunculiasis. In December 2024, 86% of all 409 villages had a source of copepod-free drinking water (e.g., borehole well). During January 2024–June 2025, national and provincial political leaders pledged support for Guinea worm eradication.

**Ethiopia**. Ethiopia reported no human dracunculiasis cases during January 2023–June 2025 (Table 2). Surveillance was conducted in 474 villages and other areas including farms and other temporary habitations. Two infected baboons were detected in 2024. In April 2024, one nonemerged worm from a baboon did not meet the case definition and was therefore not counted. In 2024, 96% of surveyed persons in areas under active surveillance knew of the rewards for reporting infected animals; in January–June 2025, 99% knew of the rewards.

Since April 2018, Ethiopia has supported villager-initiated tethering of approximately 1,900 dogs and cats in villages at highest risk for the disease. In addition, temephos is applied monthly to water sources known to be used by humans or infected animals in areas at risk for the disease.

**Mali.** Guinea worm transmission in Mali is complicated by the commercial marketing and transport of dogs for human consumption. Mali reported no human dracunculiasis during January 2024–June 2025, compared with a single case in 2023 (Table 2). In 2024, a total of 28 infected animals were reported, a 39% decrease compared with 46 in 2023. Mali reported two dog infections during January–June 2025, compared with no animal infections reported during the same period in 2024. All infected animals were in areas that were relatively inaccessible by public health personnel because of civil unrest.

In 2024, a total of 1,966 villages in Mali were under surveillance for dracunculiasis (Table 1). In 2024, 87% of persons in these areas knew about the rewards for reporting dracunculiasis; during January–June 2025, 94% knew of the rewards. All dogs in Mali are tethered during June–September, the peak Guinea worm transmission season.

**South Sudan.** South Sudan reported six human Guinea worm cases in 2024 and two in 2023 (Table 2). No cases were reported during January–June 2025, compared with two during January–June 2024. Two infected cats, one dog, and one genet (a small African carnivore) were detected in 2024, as well as 14 small carnivores, including servals, other wild cats, and civets, with nonemerged Guinea worms in 2024. No infected animals were detected during January–June 2025. Sporadic civil insecurity is a challenge to surveillance and interventions. By December 2024, a total of 2,490 villages in South Sudan were under surveillance (Table 1).

### Health Care Worker Training

GWEP activities rely heavily on public health personnel. Training for case detection, containment, reporting, and topical treatment of Guinea worm lesions, as well as education about safe water, resulted in the cumulative addition of thousands of trained health officers in the affected countries.

## Discussion

The 15 human cases of dracunculiasis in 2024 represent the third lowest annual case count ever recorded, after 13 cases in 2022 and 14 cases in 2023. Eradication progress was reviewed at the 2024 and 2025 annual meetings of GWEP managers and at unofficial meetings during the 2024 and 2025 WHAs. As a result of the detection of animal infections in 2012 and WHO’s revision of the definition of global eradication in 2023,*** WHA adopted a new resolution supporting Guinea worm eradication (WHA 78.14) in May 2025 endorsing an enhanced multipoint eradication strategy and urging stronger national commitments and political support.

### Infections in Humans

Detection of no human cases in Angola and Cameroon during January 2023–June 2025 and only one case in Mali during that period suggests that risk for transmission to humans in those countries is limited but has not been eliminated. Adequate security is imperative to achieving eradication, especially in Mali and South Sudan. Although Sudan has not detected dracunculiasis since 2002 and never detected an infected animal, the country has not yet been certified Guinea worm–free by WHO because of civil insecurity.

### Infections in Animals

Animal infections are a main challenge to dracunculiasis eradication. The existing GWEP and cooperation among its partners facilitated identification of new sources of sustained transmission in animals. Environmental contamination by infected dogs sustains transmission to humans, cats, and other dogs. Animal infections are being addressed through innovative interventions and research supported by The Carter Center, CDC, and WHO (*2*,*5*,*7*). Proactive tethering of dogs in villages at risk for the disease, application of temephos, and safe disposal of aquatic animal waste are the main strategies for preventing transmission in animals (*1*,*5*).

 Cameroon’s slow remobilization after discovery of new Guinea worm infections allowed reestablishment of indigenous transmission after cross-border transit of infected community-owned dogs from Chad. In Mali, transmission is aided by commercial marketing and transport of dogs for human consumption. In 2024, South Sudan’s expanded surveillance revealed dracunculiasis in small wild carnivores; baboons appear to be sustaining dracunculiasis transmission in Ethiopia. In Chad, animal dracunculiasis cases decreased for the fifth and sixth consecutive years, by 43% from 2023 to 2024 and by 44% from January–June 2024 to January–June 2025. 

### Limitations

The findings in this report are subject to at least two limitations. First, GWEP surveillance has recognized shortcomings, including underreporting, missed infections, and limited accessibility due to insecurity and civil unrest. Second, accurate ascertainment of the prevalence of dracunculiasis in wildlife is a challenge, although most remaining foci appear to be driven by infected dogs.

### Implication for Public Health Practice

GWEP activities led to regional improvements in health, health education, and access to cleaner water (*9*). Programs have increased the numbers of trained and experienced health and One Health officers, and village volunteers. Dracunculiasis eradication would represent a monumental public health accomplishment, having been achieved without a vaccine or administration of medications. Continued cooperation with partners and research institutions can help elucidate unusual epidemiologic characteristics of dracunculiasis in the remaining affected countries and help guide development of new interventions to reach eradication.

## Footnotes

* A dracunculiasis case is defined as an infection occurring in a person exhibiting a skin lesion or lesions with emergence of one or more worms that are laboratory confirmed by CDC as *D. medinensis*. Because *D. medinensis* has a 10- to 14-month incubation period, each infected person is counted as having an infection only once during a calendar year.

† Initially 20 countries, but the former country of Sudan officially separated into two countries (South Sudan and Sudan) on July 9, 2011.

§ Human cases are considered contained when all of the following criteria are met: 1) infected patients are identified within 24 hours of worm emergence; 2) patients have not entered any water source since worm emergence; 3) a village volunteer or health care provider has properly treated the lesion until all detectable worms are fully removed and has educated the patient on how not to contaminate water sources; 4) the containment process is validated by a GWEP supervisor within 7 days of worm emergence; and 5) all contaminated and potentially contaminated sources of drinking water are treated with temephos. The criteria for defining a contained case of dracunculiasis in a human should also be applied, as appropriate, to define containment for an animal with a Guinea worm infection.

¶ Villages under active surveillance are those with endemic dracunculiasis or that are at high risk for importation. Active surveillance involves daily searches of households by village volunteers (supported by their supervisors) for persons or animals with signs of dracunculiasis.

** 45 C.F.R. part 46.102(l)(2), 21 C.F.R. part 56; 42 U.S.C. Sect. 241(d); 5 U.S.C. Sect. 552a; 44 U.S.C. Sect. 3501 et seq.

†† An indigenous dracunculiasis human case or animal infection is defined as an infection consisting of a skin lesion or lesions with emergence of one or more Guinea worms in a person or animal with no history of travel outside their residential locality during the preceding year.

§§ CDC identifies specimens in the laboratory as *D. medinensis* by morphologic examination under a microscope or DNA sequencing assays. CDC | DPDx | Diagnostic Procedures | Other Specimens

¶¶ Refers to dogs belonging to a member of a village or community and does not refer to stray dogs.

*** The absence of adult female worms (i.e., interruption of *D. medinensis *transmission) in humans and animals for ≥3 consecutive years worldwide. Criteria for the certification of dracunculiasis eradication, 2023 update. Geneva: World Health Organization; 2023
